# Encouraging probiotics for the prevention and treatment of immune-related adverse events in novel immunotherapies against malignant glioma

**DOI:** 10.37349/etat.2022.00114

**Published:** 2022-12-27

**Authors:** Sayuri Yoshikawa, Kurumi Taniguchi, Haruka Sawamura, Yuka Ikeda, Ai Tsuji, Satoru Matsuda

**Affiliations:** Department of Food Science and Nutrition, Nara Women’s University, Kita-Uoya Nishimachi, Nara 630-8506, Japan; Tianjin Medical University General Hospital, China

**Keywords:** Glioma, oncolytic virus therapy, immune-related adverse events, gut microbiota, gut-brain-immune axis, engram, immune-related disorders, reactive oxygen species

## Abstract

Among the malignant tumors in the central nervous system (CNS), glioma is the most challenging tumor to the public society, which accounts for the majority of intracranial malignant tumors with impaired brain function. In general, conventional therapies are still unable to provide an effective cure. However, novel immunotherapies have changed the treatment scene giving patients a greater potential to attain long term survival, improved quality of life. Having shown favorable results in solid tumors, those therapies are now at a cancer research hotspot, which could even shrink the growth of glioma cells without causing severe complications. However, it is important to recognize that the therapy may be occasionally associated with noteworthy adverse action called immune-related adverse events (IRAEs) which have emerged as a potential limitation of the therapy. Multiple classes of mediators have been developed to enhance the ability of immune system to target malignant tumors including glioma but may also be associated with the IRAEs. In addition, it is probable that it would take long time after the therapy to exhibit severe immune-related disorders. Gut microbiota could play an integral role in optimal immune development and/or appropriate function for the cancer therapy, which is a vital component of the multidirectional communication between immune system, brain, and gut, also known as gut-brain-immune axis. Here, we show the potential effects of the gut-brain-immune axis based on an “engram theory” for the innovative treatment of IRAEs.

## Introduction

Glioma is the most common primary malignant tumor of the central nervous system (CNS) in adults, with about 50% of patients showing the most aggressive form, glioblastoma that may be the most advanced stage with the worst prognosis [1]. Conventional therapies, including surgery, radiotherapy, and/or pharmacotherapy, have not resulted in improvements in the survival outcomes [2]. Now, novel immunotherapies may represent a breakthrough that has radically changed the therapeutic management of numerous cancer types in oncology. There are several modalities of the immunotherapy being evaluated in patients with cancers, including peptide vaccines, oncolytic viruses, chimeric antigens receptor-T (CAR-T) cells, and immune checkpoint blockade therapy. Immunotherapy utilizes new mechanisms of the treatment, which may be also available in the field of gliomas. Given the success with many solid tumors, in fact, the potential of immune checkpoint blockade therapy, has been actively followed for the treatment of glioblastoma. However, glioblastoma lack T-cell infiltration compared with the other tumor types [3], which may limit the availability of immune checkpoint blockade for the treatment of glioblastoma [4]. In this regard, glioblastoma is thought as a type of “cold tumor” in the immune checkpoint blockade therapy [4]. Therefore, recent advances in oncolytic virus immunotherapy have generated great expectations that glioblastoma is thought as a type of “hot tumor” in the oncolytic virus immunotherapy. Oncolytic viral therapy is a well-designed immuno-therapeutic method against various tumors to kill cancer cells by means of virus replication [5], which is also an approach that involves the use of a genetically engineered virus. The virus could infect and/or replicate specifically in cancer cells [6]. The activity of the virus is dependent on the replicative potential and/or its ability to avoid antiviral responses provoked by the host [7]. The virus infection and/or the oncolysis of tumor cells may bring up an immune response which causes an adaptive antitumor response to furthermore kill the tumor cells [8]. Thus, oncolytic viral therapy could offer a dual (innate and adaptive) mechanism of antitumor responses to the induction of systemic antitumor immunity [9], which might be a novel class of therapeutic medicine in the treatment of various solid tumors including glioblastoma. Although promising, more clinical trials are needed to prove the safety and/or efficacy of the oncolytic viral therapy, as the oncolytic viral therapy could induce immense immunogenic cell death of tumor and/or normal cells [10]. Valuable oncology without triggering an excessive inflammatory response may be also needed for the safe administration of those therapies as well as the identification and/or management of immune-related toxicities. In addition, immunomodulation and/or multidisciplinary coordination are keys for the management of the toxicity produced by the novel immunotherapies.

## Possible serious outcomes from immune-related-adverse events

Novel immunotherapies such as immune checkpoint blockade therapy and/or oncolytic viral therapy have characterized a breakthrough in the oncology, which has drastically changed the therapeutic management of numerous cancer types. In fact, it has been shown that those immunotherapies could improve the survival rate in a broad range of cancers [11]. In particular, both immune checkpoint blockade therapy and oncolytic viral therapy are promising alternative to conventional therapies with a certain degree of safety and/or efficacy in the treatment of glioma [12], as the prognosis of malignant gliomas remains poor. The oncolytic viral therapy undoubtedly represents a breakthrough in glioma, which could elongate the survival [12]. However, the possibility of using such novel immunotherapies may cause undesired toxic effects in the patient, which may be associated with immune-related adverse events (IRAEs). Some cancer therapies such as radiotherapy and/or chemotherapy could induce immunogenic cell death to promote immune activation, which might be also related to features of the IRAEs. Up to 95% of patients receiving novel immunotherapy may experience the IRAEs due to immune dysregulation, which have been identified in the liver, lungs, and nervous system [13, 14]. Furthermore, myocarditis, nephritis, and even hematological adverse events have been reported [15, 16]. Most of the symptoms in IRAEs could be regulated by appropriately adding systemic glucocorticoids, which is one of the existing challenges. However, the corticosteroid-associated adverse events may remain a cause of concern for a prolonged period [17].

**Figure 1.**
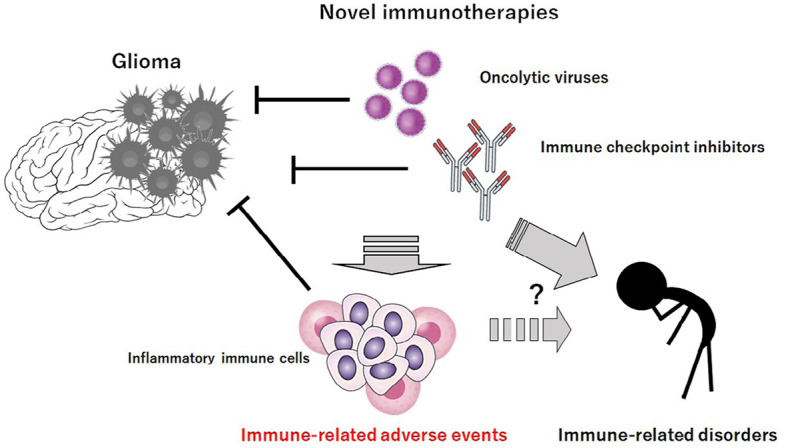
Novel immunotherapies such as “oncolytic virus therapy and/or” immune checkpoint blockade therapy could decrease the growth of glioma cells. However, these immunotherapies may be associated with unfavorable IRAEs possibly via the production of excess inflammatory immune cells. It would probably take long time to exhibit several immune-related disorders after the immunotherapies

Some immunotherapies have resulted in worse and/or even fatal outcomes, which are often associated with the development of neurotoxicity/cerebral edema and cytokine release syndrome with sepsis and/or multiple organ failure [18, 19]. In addition, treatment-related deaths have been found to be attributed to serious adverse effects such as cardiac failure, large intestine perforation, Stevens-Johnson syndrome, pulmonary embolism, and respiratory tract infections [20]. Oncolytic viruses engineered from herpes simplex virus 1 may have thymidine kinase gene which could facilitate viral replication [21]. Some compensatory mutations could compromise safety, which can be of concern [22]. Those harmfulness might be frequently related to inflammatory effects and/or off-target immune effects. Some of the IRAEs could progress into actual autoimmune diseases with autoantibodies and/or symptoms fairly undistinguished from the naturally occurring autoimmune diseases [23], in which inflammatory mitochondrial dysfunction may be regarded as the core of their pathological processes [24]. It has been implicated that accelerating cancer progression may also remain a cause of concern [25]. Probably, it would take very long time as a late onset adverse event to exhibit several immune-related disorders (Figure 1). Further clinical studies are compulsory to maximize the therapy-benefits and minimize the risks of novel immunotherapies.

## How to manage the IRAEs

The immune response activation in several organs could arise IRAEs, for which the underlying precise mechanism still remains obscure. Accumulating evidence offers that gut microbiota is associated with the occurrence of IRAEs following the treatment with immune checkpoint inhibitors, suggesting that some intestinal bacteria could effectively distinguish patients without IRAEs from patients with IRAEs [26, 27]. In addition, it has been revealed that metabolites produced from gut microbiota could stimulate several immune cells. For example, short-chain fatty acids (SCFAs) could stimulate an inflammasome via the G protein coupled receptor (GPCR) dependent mechanisms [27], which could also show a protecting effect in inflammatory reactions [28]. Thus, gut microbiota may influence on innate and adaptive immune cells, and systemic circulation may be associated with various neuro-inflammatory and psychiatric disorders with important consequences for brain behavior [29]. As the gut microbiota could modulate CNS through different pathways including this immune system, it is reasonable to consider the impact of gut microbiota on immune related disorders including IRAEs, where inflammation may play a key role. In fact, gut microbiota interacts with the enteric and CNS through complex bidirectional signaling along the gut-immune-brain axis [29]. In addition, increasing data have been indicating the complicated relations between gut microbiota, immunity and CNS [30]. Recently, we have described that immunological memory named “engrams” could restore the disease situation/condition [31]. Based on these concepts, several therapeutic strategies for IRAEs could be applied to the modification of gut microbiota.

One method for the action that may effectively impact on the composition of gut microbiota is probiotics, which may often include components of daily food products such as yogurt [32]. Importantly, many studies have demonstrated beneficial effects on patients with immune disorders by the probiotic administration. For example, it has been reported that *Bifidobacterium* could mitigate an immunopathology of IRAEs by the alteration of gut microbiota composition in the systematic regulatory T cell (Treg)-dependent manner [33]. Similarly, the enormous potential of fecal microbiota transplantation (FMT) might be a promising therapeutic strategy to alter and/or repair the composition of gut microbiota [34]. It has been shown that FMT can effectively cure patients with IRAEs [35]. A favorable microbiota might have the potential to ameliorate IRAEs [36]. Therefore, manipulating the gut microbiota by FMT might be expected to reduce various immune-related adverse reactions [37]. Whereas the usage of gut microbe-targeted therapies including probiotics, prebiotics, and/or FMT could promote immune response, different gut microbiota could be also associated with the resistance to immune checkpoint inhibitors [38]. The gut microbiota has emerged as a key regulator of not only systemic immune regulation in immunology but also in oncology.

## Gut microbiota may also affect the development of glioma

**Figure 2.**
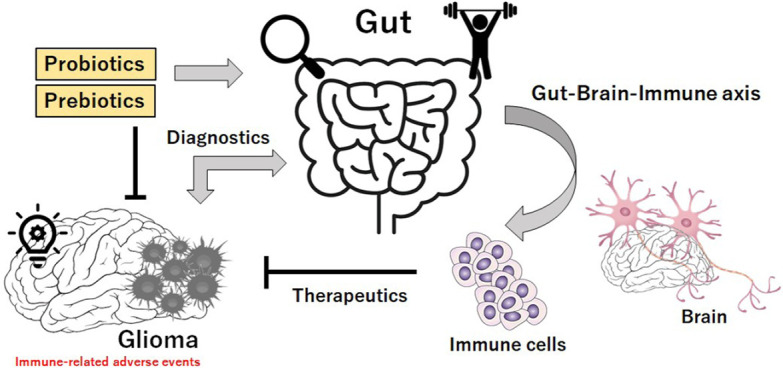
Schematic illustration implying that gut microbiota could be considered for theranostic applications. There might be a link between the gut microbiota and the development of glioma, and glioma can induce the alterations in gut microbiota. In addition, gut microbiota could possibly play a key role via the gut-brain-immune axis in the effectiveness of novel immunotherapies against glioma. Theranostics may be a combination of the terms therapeutics and diagnostics. Therefore, gut microbiota could be a significant theranostic tool for the treatment of glioma

Several evidences have shown a solid connection between gut microbiota and CNS in pathological and/or physiological situations. The composition of gut microbiota could affect mood, behavior, and cognition via the production or modulation of several neurotransmitters such as dopamine, serotonin, norepinephrine, and/or gamma-aminobutyric acid [39]. These neurotransmitters in turn could modulate the function of brain [40]. In addition, neurotransmitters have been shown to be linked with the uncontrolled proliferation of cancer cells [41]. Mechanistically, the phosphatidylinositol 3 kinase (PI3K)/protein kinase B (AKT) signaling pathway has been revealed to be possibly activated under the stress conditions for the proliferation of cancer cells [42]. In fact, several studies have documented a role for the gut microbiota in the development of certain tumors. For example, antibiotic treatment through a gut microbiota-immune-microglia axis has concerned with a pro-inflammatory pathway leading to the increased growth of glioma [43]. An altered gut–immune–brain communication might contribute to bring a tumor-tolerant CNS microenvironment, which helps glioma development [43]. In addition, it has been reported that gut microbiota could impact on microglial phenotype in brain [44], with a shift toward a more immune-suppressive and/or tumorigenic condition [43, 44]. Furthermore, gut microbiota may be related to the growth of various tumors, suggesting that the identification of gut microbiota could be a tool for the diagnosis of brain tumors [45] (Figure 2). Through the reduction of cytotoxic NK cells, chronic treatment with antibiotics could stimulate the growth of glioma [43, 46]. In addition, evidences have demonstrated the role of gut-dysbiosis in promoting inflammatory responses and/or cancer initiation, which is a remarkable change in the balance of bacterial ecosystem that could favor chronic inflammation and/or immunosuppression [47]. It has been hypothesized that there is a link between the gut microbiota and the development of glioblastoma through the immune regulation. In fact, the metabolic regulation of glioma has been shown on the environment through gut microbiota, which could impact on the prognosis of patients [48]. Possibly, altered metabolites from gut microbiota might affect systemic and/or CNS immunity. In fact, alterations of the microbiota-composition could contribute to the growth of glioma by weakening the immune condition of brain [43, 49]. Another study has demonstrated that glioma can also induce alterations in the microbiota [50]. Moreover, a diversity of the gut microbiota with glioma patients has been shown to be different from that of healthy subjects [45, 50]. All together, these studies demonstrate that the bidirectional axis between the gut and the brain may be a determinant of the glioma biology. Therefore, gut microbiota could be a significant theranostic tool in glioma (Figure 2).

## Innovative praxis with gut-brain-immune axis for the potential treatment of immune-mediated inflammatory disorders

Glioma development could lead to gut-dysbiosis in a mouse-model, which could promote inflammation [50]. In addition, the gut-dysbiosis could further accelerate glioma progression [49]. IRAEs are practically similar to the condition from clinically significant inflammatory toxicities. It is well known that the immune-mediated inflammatory disorders are considered relative contraindication to the novel immune therapy due to the risk of provoking flares [51]. In this context, appropriate gut-directed interventions such as diet, nutritional supplementation, and/or fecal transplantation could calm down the inflammatory response and/or improve the course of immune-mediated inflammatory disorders [52]. The dynamic residents of microbes in gut may play a fundamental role in managing the host immune-physiology. In addition, recent advances have emphasized the importance of the gut microbiota in neuro-immune development with significant implications for the onset and/or the development of neurodegenerative disorders [53]. Some reports have shown that the dysbiosis of gut microbiota could exacerbate many immune-related neurodegenerative disorders [54]. Alterations in the composition of gut microbiota with an increased number of potentially pathological organisms might also play a prominent role in the pathogenesis of those disorders. For example, amyotrophic lateral sclerosis (ALS) patients often exhibit differences in their gut microbial communities compared with the paired controls [55]. Besides, a more dysbiosis in gut has been shown to exacerbate the symptoms of ALS patients [56]. Emerging evidence also links the gut dysbiosis to the immune-mediated chronic neuroinflammation [57].

Associations of engrams are thought to determine the condition either health or inflammatory diseases by arrangements. Consequently, the immunological engrams could restore the initial inflammatory disease-condition, if rebooted [58]. Maintenance of the memory might be achieved by a meta-plasticity mechanism that raises the change in neurons within an engram. In short, the brain could possess inflammatory responses as an information of pathological images in brain. Some engrams could activate and/or aggravate the conditions of immunological disorders [59]. Therefore, emptying the bad pathological memory of engrams might be encouraging for the prevention and/or the treatment of certain immunological disorders. We believe it might be possible to clear out the memory of engrams without any damage in brain. This is the point for the therapeutic interventions to the immunological disorders. In one conceivable approach, the elimination and/or oblivion of bad memories with engrams might be achieved by synaptic removal with an action of microglia [60]. Microglia could make this synapse purging as a mechanism for memory maintenances [60, 61]. In addition, it has been reported that microglia is intensely related to the synapse density, learning, and memory [62]. There are significant associations between gut microbiota and demyelination by the microglia in brain, suggesting that the crosstalk between gut-microbiota and brain-microglia could play a key role for clearing out the engrams [63]. In fact, the destruction of the homeostasis in the gut-brain axis could lead to cognitive impairment and memory decline [64].

Gut microbiota could also regulate the reactive oxygen species (ROS) levels to preserve the brain healthy [65], which might skew the function of microglia with the oxidized mitochondria within glial cells [66]. Alteration of microglia, oxidative stress, and/or inflammatory factors are all known to limit neuroplasticity in CNS [67]. Consequently, certain gut microbiota with the capability of producing proper levels of ROS for healthy brain could probably improve and/or attenuate the symptoms of immune disorders by clearing out the pathological memory of engrams via the alteration of microglia in brain. Studies had proven that some species of bacteria can produce catecholamines and/or acetylcholine, which could contribute to the response of sympathetic nerve [68]. Interestingly, it has been shown that activation of the vagal nerve may enhance the hippocampus-dependent memory via the non-competitive antagonist of nicotinic-acetylcholine receptors [69]. In addition, scopolamine treatment may significantly increase impairments in memory and cognitive function associated with increased level of pro-inflammatory cytokines [70]. On the contrary, the stress-induced elevation in acetylcholine level may act to improve the inflammatory response [71]. Convincing evidence has demonstrated the roles of gut microbiota in the pathogenesis of Alzheimer’s disease and Parkinson’s disease, which is partly mediated by modified microglial activity in the brain [72].

**Figure 3.**
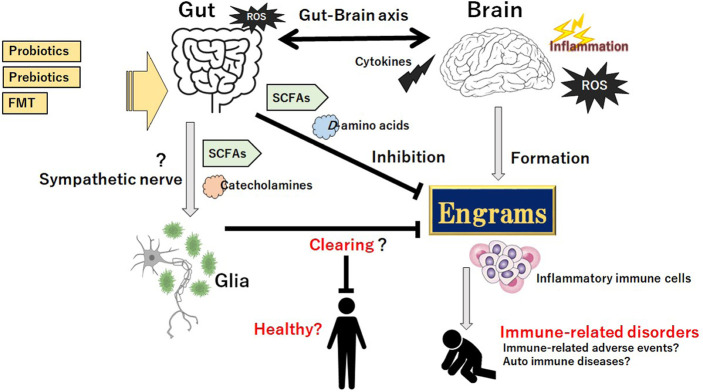
Gut microbiota could assist the favorable achievement against the progression of immune-related disorders possibly including IRAEs and/or auto immune diseases by clearing the engrams, which might be accomplished by the production of SCFAs, certain D-amino acids, and/or various catecholamines, in part through sympathetic nerve pathway to the glia cells in CNS for clearing the pathogenic memory of engrams. Probiotics, prebiotics, and FMT might have potential to change the composition of gut microbiota for the treatment of immune-related disorders. Arrowhead suggests stimulation whereas hammerhead indicates inhibition. Note that some critical actions such as ROS-production and/or cytokine-induction in the gut-brain axis have been omitted for clarity

The signaling mechanism behind the communication of gut-brain axis should be of particular interest when considering the therapeutic design. The brain could modulate the gut function in part through the autonomic nervous system [73]. Remarkably, innovative treatments for the immune-neurodegenerative disorders including schizophrenia are emerging [31, 74]. As already mentioned above, one method for the action that might effectively impact on the composition of gut microbiota is FMT. By transplanting the healthy gut microbiota, it may be possible to amend the efficacy of gut microbiota for the treatments of neurodegenerative disorders [74]. Similarly, administration of prebiotics and/or probiotics might be applicable to prevent and/or cure for the immune-neurodegenerative disorders. The prebiotics are particular plant fibers, which may stimulate the growth of healthy bacteria in gut. The probiotics usually contain specific alive organisms, which directly increase to the population of healthy microbes in gut. Though the alteration of gut microbiota, these technologies could be an innovative treatment for the immune-related disorders including IRAEs in novel immunotherapies against glioma, where something as simple as an every-day diet may proffer noteworthy therapeutic potential (Figure 3).

## Future perspectives

Although severe IRAEs may remain sporadic at present, they could become life-threatening if not handled properly. In fact, the pathological features of a severe liver-injury associated with the administration of the anti-PD-1 antibody have been reported in a patient with glioblastoma [75]. It will be important to recognize that the gut microbiota may be a key contributor to the prevention, development, treatment, and management of several immune-related diseases including IRAEs. New strategies will become available with increased knowledge on the gut microbiota for a variety of immune disorders. In fact, evolving evidence suggests that dysbiosis of gut microbiota might also play important roles in the occurrence and/or development of autoimmune diseases, which could indicate potentials for the successful treatment of an autoimmune disease with the manipulation of gut microbiota [76, 77]. The different populations of gut microbes could be considered beneficial in some cases and/or potentially dangerous at the same time to the host. Only advancements could guide the future perspectives in this field. The progress of this field would have enabled a substantial step closer to understanding the pathophysiology of immune diseases through unravelling the intricate networks of the immune system, gut microbiota, and gut-brain-immune axis. Also, this is an exciting development for the field of brain oncology. Huge flexibility of the microbiota might imply potential for a new frontier toward precision medicine.

## Abbreviations

CNS: central nervous system
FMT: fecal microbiota transplantation
IRAEs: immune-related adverse events
ROS: reactive oxygen species

## Declarations

### Copyright

© The Author(s) 2022.
